# Role of Innate Immunity in Initiation and Progression of Osteoarthritis, with Emphasis on Horses

**DOI:** 10.3390/ani11113247

**Published:** 2021-11-13

**Authors:** Juan Estrada McDermott, Lynn Pezzanite, Laurie Goodrich, Kelly Santangelo, Lyndah Chow, Steven Dow, William Wheat

**Affiliations:** 1 Department of Clinical Sciences, Colorado State University, Fort Collins, CO 80523, USA; juan.estrada_mcdermott@colostate.edu (J.E.M.); lynn.pezzanite@colostate.edu (L.P.); laurie.goodrich@colostate.edu (L.G.); lyndah.chow@colostate.edu (L.C.); steven.dow@colostate.edu (S.D.); 2 Department of Microbiology, Immunology and Pathology, Colorado State University, Fort Collins, CO 80523, USA; kelly.santangelo@colostate.edu

**Keywords:** equine, osteoarthritis, innate immunity, macrophages

## Abstract

**Simple Summary:**

Osteoarthritis (OA) is a common condition affecting horses and humans. The role of the innate immune system in the pathogenesis and progression of OA has been increasingly recognized in recent years. This review examines the function of the innate immune system in perpetuating inflammation seen in OA, drawing important insights across species from animal models of OA and humans, and discusses therapeutic immune modulatory options currently available to manage OA.

**Abstract:**

Osteoarthritis (OA) is a common condition with diverse etiologies, affecting horses, humans, and companion animals. Importantly, OA is not a single disease, but rather a disease process initiated by different events, including acute trauma, irregular or repetitive overload of articular structures, and spontaneous development with aging. Our understanding of the pathogenesis of OA is still evolving, and OA is increasingly considered a multifactorial disease in which the innate immune system plays a key role in regulating and perpetuating low-grade inflammation, resulting in sustained cartilage injury and destruction. Macrophages within the synovium and synovial fluid are considered the key regulators of immune processes in OA and are capable of both stimulating and suppressing joint inflammation, by responding to local and systemic cues. The purpose of this review is to examine the role of the innate immune system in the overall pathogenesis of OA, drawing on insights from studies in humans, animal models of OA, and from clinical and research studies in horses. This review also discusses the various therapeutic immune modulatory options currently available for managing OA and their mechanisms of action.

## 1. Osteoarthritis: General Review

### 1.1. Epidemiology

Osteoarthritis (OA) is one of the most common causes of joint disease in many species, including humans and horses. OA in humans has a huge medical and economic impact [1,2]. Approximately 30.8 million people in the United States (13.4% of the adult population) suffer from OA, resulting in $185 billion in medical costs incurred each year [3]. Similarly, in horses, OA is considered the most common cause of lameness, accounting for approximately 60% of the caseload presenting for soundness evaluations [4]. McIlwraith et al. reported the direct and indirect medical costs associated with OA to be between $3000–15,000 per horse each year in the U.S. alone [5]. Specific populations of horses may also suffer from higher rates of OA, with up to 66% of Thoroughbred racehorses developing this disorder, resulting in pain and significantly shortened career-spans [6,7].

### 1.2. Pathogenesis

Development of OA is associated with multiple predisposing factors such as age, acute trauma, and/or irregular or repetitive overload of articular structures. Each of these conditions can produce progressive deterioration of cartilage surfaces, as well as soft tissue and subchondral bone remodeling. The chronic form of this disease has classically been considered a non-inflammatory degenerative condition, but as recent manuscripts suggest [8,9], the pathophysiology is much more complex than a simple “wear and tear” or aging phenomenon and immune events in OA involve not only articular cartilage but also the synovium, adipose tissues, and subchondral bone [8,9].

Understanding of the causative mediators and the molecular, cellular, and tissue relationships driving progressive intra-articular degradation in OA remains incomplete, and repair or regeneration of full-thickness articular cartilage defects poses a particular challenge in the orthopedic field. Indeed, the degree of overall joint deterioration may not simply be due to the type of initial damage (i.e., microdamage versus a single catastrophic trauma) but rather the extent and progression of the intraarticular milieu that drives subsequent pro-inflammatory and catabolic states. As such, OA in horses can be viewed as a multifactorial condition that includes low-grade, chronic joint inflammation resulting from, and continually propagating, physical damage of articular surfaces and joint degeneration as a whole [9,10,11]. This recent work highlights the hypothesis that innate inflammation is driven to a large extent by synovial macrophages, blurring the demarcation between definitions of inflammatory versus degenerative diseases [9,10,11].

Research into immune mechanisms in OA has included characterization of inflammatory cytokines, cellular infiltrates, and resident tissue responses in the synovium and cartilage [9,12,13,14]. As OA is primarily diagnosed radiographically, frequently after irreversible damage to the joint has occurred, there remains a critical need to improve understanding of factors contributing to early disease progression. Continued efforts to understand earlier stages of OA are relevant and warranted, as inflammation and micro-damage exist prior to radiographical manifestations of disease such as osteophyte formation or joint space narrowing [12,13,14].

This review will examine the role of the innate immune system in the overall pathogenesis of OA, from early to late events, and will draw on a comparative perspective to literature derived from laboratory animals and humans for implementation in equine clinical practice. Disease-modifying interventions are also discussed from the perspective of immune modulation and their role in slowing OA progression.

## 2. Innate Immunity and OA: Lessons from Animal Models and Clinical Practice

### 2.1. Pattern Recognition Receptors and Immune Cells in Joints

**Figure 1:**
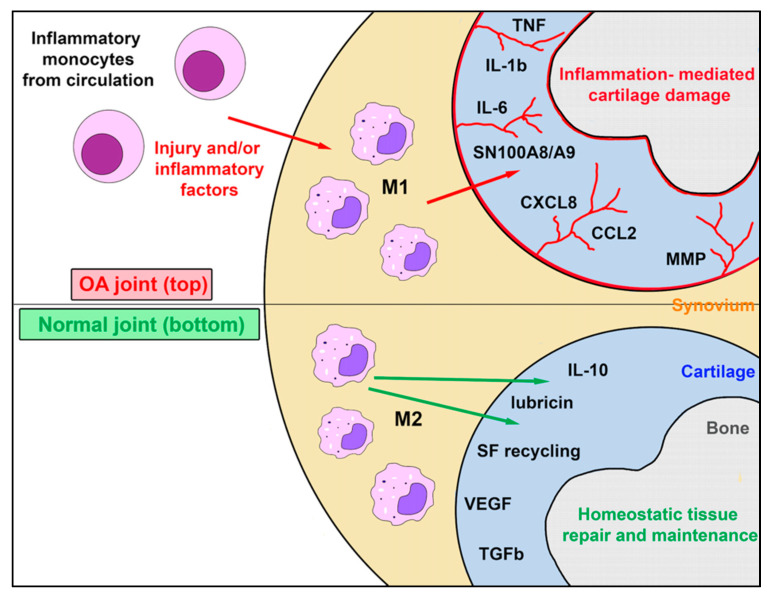
Role of synovial resident macrophages and inflammatory macrophages in joint health and osteoarthritis. This figure illustrates the complex interaction between endogenous self-renewing synovial cell macrophages, which exist as M2 cells in the healthy joint, and the inflammatory macrophage M1 population recruited from inflammatory monocytes in blood in response to chemokines produced during joint inflammation occurring during the progression of OA. While M1 macrophages may assume an M2 phenotype as inflammation subsides, endogenous M2 macrophages typically do not generate M1 macrophages. The factors secreted by synovial M1 and M2 macrophage populations in health and disease are complex and largely distinct. Abbreviations: tumor necrosis factor (TNF); interleukin 1Β (IL-1Β); interleukin 6 (IL-6), Myeloid protein SN100A8/A9; C-X-C Motif Chemokine Ligand 8 or Interleukin-8 (CXCL8); (CCL2); matrix metalloproteinase (MMP); interleukin 10 (IL-10); synovial fluid (SF); vascular endothelial growth factor (VEGF); transforming growth factor B (TGF-B).

Although understanding of OA pathogenesis is still evolving, innate immune cells, particularly myeloid cells, play a role in regulating and perpetuating low-grade inflammation that characterizes OA (Figure 1). Histologically, synovial inflammation in OA is characterized by transient and/or cyclical hyperplasia of the synovial lining cells accompanied by inflammatory cell infiltration. This population consists predominantly of macrophages and smaller numbers of T and B cells, mast cells, and natural killer (NK) cells [15,16,17,18].

The innate immune system is classically triggered by host responses to pathogen-associated molecular patterns (PAMPs) induced by interactions with invariable pattern-recognition receptors (PRRs) on synovial joint immune cells such as neutrophils, macrophages, monocytes, and dendritic cells (DC). PRRs are comprised of a family of cell surface, endosomal and cytosolic receptors, including Toll-like receptors and NOD-like receptors [19]. Activation of PRRs within tissues such as the joint leads initially to rapid-onset inflammatory responses, followed later by initiation of adaptive immune responses and finally by healing responses in the case of tissue injuries.

In addition to invading pathogens, PRRs also recognize various endogenous “danger signals,” known as damage associated molecular patterns (DAMPs), triggered from cell or tissue damage. Non-infectious DAMPs (e.g., cartilage degradation products) can activate macrophages and DCs, including inflammasome activation, initiation of cellular pyroptosis, and other unfolded protein responses that induce inflammatory, metabolic, and adaptive immune pathologies [20,21]. The list of known DAMPs is rapidly growing and beyond the scope of this review. Studies have shown that cartilage injury results in the release of DAMPs within the joint, including breakdown products from fibronectin and hyaluronan [22,23,24].

### 2.2. Macrophages and OA

Macrophages are central players in host defense and are distributed in almost all tissues, having unique functions in each organ depending on the specific microenvironment that influences their functional properties. In addition to their more familiar functions as proinflammatory, scavenger, antimicrobial, and antitumor defense effector/mediators, macrophages also function as immune modulators, promoting anti-inflammatory and tissue repair processes [25,26,27,28,29]. Given their central role in OA, a clinical approach targeting activated macrophages at an earlier stage of OA may serve to inhibit or slow the progression of disease [30,31].

Tissue macrophages such as alveolar macrophages and Kupffer cells are derived from the yolk sac during early embryogenesis and serve as a self-renewing population of tissue macrophages throughout life. It is likely that synovial macrophages are also a unique tissue specific population of self-renewing cells, which have an important role in maintaining articular homeostasis. Inflammatory monocytes by contrast reach the joint following release from the bone marrow and differentiate into short-lived inflammatory macrophages in synovial tissues (Figure 1). Macrophages in tissues are classified as proinflammatory (M1) or anti-inflammatory (M2) cells [32]. In rheumatoid arthritis, the majority of M1 macrophages are bone marrow-derived and are recruited to and differentiate within the joint. In vivo heterogeneity varies between M1 and M2 populations have been identified using next-generation sequencing technologies and transcriptomic analysis [9]. The rationale for having seemingly opposing systems is to achieve immune homeostasis via a proper balance of M1 and M2 responses, thereby maintaining a tightly controlled immune environment capable of optimal protection while preventing host tissue destruction. This is a very important consideration as macrophages are the more numerous immune cells in the synovium and further understanding is paramount before they can be exploited for therapeutic purposes [9,25,33].

M1 macrophages typically have multiple functions and are responsible for the release of molecules that can drive inflammation. M1 macrophages are differentially stimulated by NK cells and NK T cell secretion of IFNγ, by various TLR agonists such as bacterial lipopolysaccharide (LPS), or growth factors such as granulocyte-macrophage-colony-stimulating factor (GM-CSF). M2 macrophages, on the other hand, are functionally considered to be more anti-inflammatory and viewed as mediating an opposing immune regulatory response that counters proinflammatory M1 responses and abrogates host tissue destruction. M2 macrophages are stimulated by IL-4 and IL-13 secreted primarily by Th2 T cells, mast cells and, more recently discovered, by basophils—to produce mostly anti-inflammatory cytokines such as IL-10, IL-1ra, TGFβ, and arginase-1 (Arg-1) [27,34]. Under a homeostatic state, most macrophages display the M2 phenotype to maintain tissue surveillance and protect against metabolically derived oxidative conditions and inflammation [35]. Specifically, CD206^+^ M2 macrophages produce IL-4, IL-10, and MMP-12, which reduces neutrophil influx to the joint and can counter the inflammatory response and catabolic effects seen in OA [9,10,25,26,28,29,36,37,38,39,40,41,42]. 

When activated, CD80/86^+^ M1 macrophages secrete high levels of proinflammatory cytokines such as IL-1β, IL-6, IL-8, IL-12, TNFα, and alarmins; they also induce Th1 adaptive immune responses [25]. These synovial macrophages, in turn, activate the production of harmful molecules such as matrix metalloproteinases (MMP-1, MMP-3, MMP-8, MMP-9, MMP-13) from other innate immune system cells and synovial fibroblasts, causing generation of DAMPs from extra cellular matrix (ECM) degeneration [25]. Additionally, pro-inflammatory cytokines stimulate synthesis of PGE_2_ by cyclo-oxygenase-2, microsomal PGE synthase-1, and soluble phospholipase A_2_ [1,25] This is associated with production of nitric oxide (NO) by nitric oxide synthetase, reactive oxygen species (ROS), 5-lipoxygenase and leukotriene B_4_ [25]. This pro-inflammatory environment also favors the activation of neuropeptides, such as substance P, that increase inflammation and result in joint pain [1,25].

Over-production of M1-derived cytokines, growth factors, various proteases, and oxidizers in inflamed synovium can heavily contribute to the initiation and progression of OA via DAMP-driven cellular pyroptosis [25,43,44,45]. DAMP activation of synovial macrophages in OA produces ROS that may incite the Nod-like receptor protein 3 (NLRP3) component of the caspase-1-activating inflammasome, mediating secretion of pro-inflammatory cytokines IL-1β, IL-18, and TNFα [25]. DAMPs identified in this process include degradation products from hyaluronidase, hydroxyapatite (HA) and basic calcium phosphate (BCP) crystals from cartilaginous calcification [25]. Although inflammasomes in veterinary species have not been well characterized, recent work has demonstrated that equine peripheral blood mononuclear cells (PBMCs) normally secrete IL-1β in response to well-known inflammasome activators of NLRP3 [43,44,45]. The key transcription factor countering the resulting oxidative stress is nuclear factor E2-related factor 2 (Nrf2), which protects against oxidative stress and tissue damage. Accordingly, future research into the signaling mechanisms transducing the Nrf2-mediated transcriptional program may serve to develop novel therapies for OA [46,47,48,49,50].

It should be noted that, in vivo, macrophages do not adhere to strict dichotomous phenotypes, but rather express plasticity across the spectrum between M1 and M2, capable of signaling for either inflammation or healing depending on tissue milieu [25]. For example, availability of various DAMPs within the joint microenvironment, their anatomical origin, and the tissue in which they are located, may influence macrophage phenotype, making it difficult to ensure that all CD86^+^ macrophages exclusively perform M1 functions and that all CD206^+^ macrophages exclusively assist in healing and resolution of inflammation. Thus, caution should be taken when describing the overall pro- vs. anti-inflammatory status within a locale [9,26]. Furthermore, studies involving murine models of macrophage depletion have reported mixed results, suggesting that this therapy might vary greatly between tissues and might not always be beneficial as all populations of myeloid cells are affected, including DCs and neutrophils [9].

### 2.3. Value of Equine Models for OA Research

Many factors can influence the outcome of data acquired from the various animal models of OA, such as overall intrinsic species and strain variation, age, sex, housing, time of intervention, stress levels, and activity. These are important considerations that must be taken into account when assessing the outcome of any project involving knowledge gathered regarding the predisposition, cause, and ultimate therapeutic success of a given research approach [51]. In vitro models are affordable and make it easier to control many variables at the same time, but the bi-dimensional model does not fully represent what truly happens in the joint. Three-dimensional models, including explants, scaffold-based and scaffold-free systems, provide a more similar environment to the joint and its interaction with the cells while allowing more variable control. In vivo models, especially large animals, remain a more viable option due to anatomic similarities and naturally occurring OA [52].

Although data from murine models is very informative and valuable in biomedical research, the variance in the programmed genomic responses to acute inflammatory responses and their significant anatomical differences might not make them the best translational option for human or equine patients [53]. From an anatomical perspective, the horse model of OA most closely resembles humans with regard to articular cartilage thickness [54]. In addition, horses suffer from OA frequently and spontaneously, providing veterinarians with significant experience in treating the condition. The equine carpal chip model of OA represents a predictable model with which to test novel approaches such as immunomodulating therapies like mesenchymal stromal cells (MSCs) [55]. Accordingly, horses are one of the best animals to use as a model for human OA, due to similarities in many of the joints’ movement and comparable cartilage and subchondral bone thicknesses [56,57].

### 2.4. Equine Innate Immune System and OA

Of the structures present in the joint, the most relevant regarding its capacity to mount an inflammatory response is the synovium. The cells that are recruited and react to post-traumatic osteoarthritis are components of the innate immune system and are mainly resident macrophages. Destruction of cartilage matrix by MMPs will result in the immediate production of IL-1β and TNFα, and these powerful pro-inflammatory cytokines can perpetuate the inflammatory cascade and pain in the joint [37,58]. Although there is a strong correlation between obesity and chronic inflammation in humans and mice [59], such a comparison has not been made for horses and it is likely that post-traumatic injury is the more common cause of OA in horses.

Synovium and innate immunity—The synovium is the major site of articular inflammation in OA and is often marked by hyperplasia of the synovial lining cells coupled with infiltration of inflammatory cells consisting mostly of macrophages and smaller numbers of other cells [15,16,17,18]. The macrophage population in the synovium is classified into two groups: 25% of the cells are type A synovial cells (macrophages) and 75% of the cells are type B synoviocytes (fibroblast-like) [12]. Also, macrophages can be found in synovial fluid and can account for 70% of the total cells in a non-inflamed joint, with these numbers increasing up to 90% in an inflammation model for repeated arthrocentesis [13]. Thus, immune cells constitute a significant percentage of all the cells in joint tissues and synovial fluid.

Cartilage and innate immunity—Mature equine cartilage predominantly contains chondrocytes, including only a very small population of progenitor cells. Cartilage is a firm yet smooth, lubricated, and almost frictionless surface that enables normal joint function and depends on the synovium for lubrication. It is important to consider that cartilage is an aneural and avascular tissue, depending directly for their metabolic support on factors present in synovial fluid. Cartilage also reacts to inflammatory reactions triggered in the synovium. It is also important to note that cartilage degradation products in synovial fluid, as well as micro-fissures in articular tissue, are often present before any degeneration can be detected using current imaging technology. It has been speculated that early cartilage degradation events may drive the development of inflammation within OA synovium, which happens through the activation of resident macrophages through DAMPS favoring the release of TNFα and IL-1β that will, in turn, increase the MMPs production by the chondrocytes [10,13].

Subchondral bone and innate immunity—Subchondral bone differs significantly from cartilage, as it is heavily vascularized, allowing for major tissue turnover and the ability to remodel to adapt to mechanical loads. Inflammation of the subchondral bone can lead to the production of angiogenic factors and local MMP production, which is thought to stimulate cartilage degeneration and the formation of osteophytes [10,60,61]. 

## 3. Immunomodulatory Therapies in Equine Practice

### 3.1. Biological Therapies

Mesenchymal Stromal Cells—Mesenchymal stromal cells (MSCs) possess immunomodulatory properties and have been used in horses to address tendon and ligament injuries, OA, laminitis, and equine asthma [29,62]. When treating degenerative joint disease, despite clinical improvement and return to work in 78% of horses, many questions remain regarding optimal dose, tissue source, and timing of administration. The equine model thus becomes useful to tackle these issues prior to translation in people. Recently, the safety and potency of autologous versus allogeneic bone marrow-derived mesenchymal stromal cells (BMDMSCs), have proven to be remarkably similar in their capacity to regulate inflammation in the musculoskeletal system of the horse [56]. This also opens the possibility of using MSCs as an “off the shelf” treatment through the creation of MSC banks [63,64]. Differences in MSC activity derived from various tissues may affect their immunosuppressive capability and could have clinical relevance as specific immune problems could be treated by MSCs from a particular tissue [65].

The ability of MSCs to elicit an anabolic signaling cascade in the joint and their immunomodulatory capacity has made them the subject of intense study as a therapeutic option for OA. Overall, MSCs can modify the inflammatory response through cell-to-cell communication or through a myriad of biologically active cell-free substances, including many chemokines and well-known anti-inflammatory cytokines like IL-6, IL-10 and TGF-β. MSCs affect cells from both the adaptive and innate immune systems, especially macrophages and T helper cells, and augment their critical role in suppressing inflammation and modifying pain during OA [66,67,68,69]. It has also been demonstrated that MSCs target T lymphocytes, affecting their pro-inflammatory activity by reducing their replication, recruitment of, and conversion to regulatory T cells (T_reg_) and promote apoptosis of the activated cells [70,71,72,73]. Murine models suggest that, once inside the joint, MSCs might undergo apoptosis quickly but still manage to affect macrophages and mediate a switch towards M2, favoring the propagation of anti-inflammatory responses [74]. Also, priming of the MSCs with pro-inflammatory cytokines favors the production of immunomodulatory molecules. In vitro stimulation of the MSCs with IFNγ prior to intra-articular injection may improve their therapeutic potential by “licensing” the cells to produce cartilage anabolic and angiogenic factors [75,76]. 

An improved understanding of MSCs has shown that, when injected directly into the joint, these cells do not promote direct healing of the cartilage but rather act through a paracrine action to produce clinical improvement [72,73,74,75]. However, ongoing research investigating enhanced effects when using scaffolds and pro-chondrogenic molecules will be paramount for improved therapy [77]. Recent studies using autologous platelet-enriched fibrin scaffolds with and without MSC showed that the addition of MSC did not improve the effects of the scaffold, which otherwise produced reasonable repair of full-thickness cartilage defects [78]. In contrast, injection of chondrogenic MSCs combined with plasma significantly improved lameness, decreased joint effusion, lowered GAG glycosaminoglycan concentration and increased viscosity of the synovial fluid, with a gross improvement of the cartilage appearance upon postmortem examination [79]. 

The paracrine activity of MSC also allows the use of their secreted factors, including exosomes, rather than the cells themselves, for therapy [80,81]. The culture methods for MSC may also significantly impact their in vivo function. For example, culturing MSC in fetal bovine serum (FBS) may alter their capacity to adapt and survive in the equine joint, though more recent studies have challenged this finding [26,82]. 

Platelet rich plasma (PRP)—Platelets are concentrated from blood, and this process results in activation through centrifugation, the platelets release a myriad of growth factors and cytokines that play a pivotal role in inflammation and tissue healing, with a direct effect on progenitor cells of diseased tissues. Although PRP is easier to produce than other biologics, the variability observed between product preparations in this process can result in inconsistent results [83,84]. Despite this, some of these PRP preparations with lower lymphocyte counts have resulted in a better clinical outcome when compared to hyaluronic acid as a commonly used treatment, in addition to steroids, for chronic pain in the joint [85,86]. 

Interleukin 1 (IL-1) receptor antagonist protein, a component of autologous-conditioned serum that neutralizes the negative effects of IL-1 by increasing IL-1rα and IL-10, elicits clinical improvement in study animals, but does not lead to regeneration of damaged tissues. There is also considerable variation in the cytokine composition depending on the preparation and species used, and still needs to be compared to other non-biological treatments [6]. 

Autologous Protein Solution is an autologous blood substituent product that requires a short (20 min) incubation of cells isolated from blood and has had produced positive results in horses with naturally occurring OA. However, it should be noted that only cases having mild radiographic and clinical signs with a short follow up (14 days) were evaluated in these studies [6]. Another study in dogs demonstrated improvement in pain levels and lameness at 12 weeks after injection. The APS contains a high autologous concentration of IL-1rα and soluble TNF receptors that will directly affect the binding of IL-1β and TNFα to target cells in the joint [87]. 

Bone marrow aspirate concentrate—Since macrophages are the most common immune cell type present in inflamed synovial tissue, they are known to be a considerable source of inflammatory and degenerative mediators of OA. Intervention strategies that modify synovial macrophages might be sufficient to alleviate OA symptoms and prevent progression [9]. It has been demonstrated that M1 polarized macrophages will inhibit chondrocyte differentiation in vitro and OA has been associated with an imbalance of M1 over M2 polarized macrophages in synovium and peripheral blood [88]. The use of bone marrow mononuclear cells (BMNCs), as reported by Menarim et al., proposes a promising therapeutic option. BMNCs consists of a population of ~50% naïve macrophages and approximately 25% of hematopoietic progenitors polarized towards the M2 subtype; this favors healing, apparently through the production of IL-10, and results in clinical improvement. It has also been suggested that some of the anti-inflammatory effects of MSCs can be attributed to precocious myeloid differentiation at the expense of self-renewal due to chronic inflammatory stimuli, as they do not produce high quantities of IL-10 themselves [26,40,89,90,91]. 

### 3.2. Gene Therapy

While beyond the scope of this review, gene therapy utilizing various viral vectors has been attempted in vitro and in vivo with differing rates of success. The optimization of this therapy with the use of self-complementary adeno-associated viral (scAAV) vectors has greatly enhanced the time for the expression of proteins like IL-1ra, demonstrating therapeutic levels for 6 to 8 months in some studies. A second study successfully demonstrated that IL-1ra re-dosing with the AAV vector was possible if a different serotype of AAV was used to avoid the effects of the neutralizing antibodies [92,93].

### 3.3. Corticosteroids 

The role of corticosteroids and their potential to downregulate macrophage mediated immunomodulation deserves further discussion. The local use of corticosteroids in equine joints, which is a common intra-articular therapy, might have the detrimental capacity to reduce the activity of the innate immune system, thereby shifting away from M2 macrophage polarization and the production of IL-10, prostaglandins (PGE_2_), and other anti-inflammatory cytokines. These biomarkers play a very important role in the resolution of inflammation, further impairing the capacity of the joint to achieve homeostasis and prolonging the duration of OA [40,94].

### 3.4. Other Therapeutic Strategies

The close relationship of subchondral bone and cartilage as a synergistic unit has been well-studied, revealing intimate crosstalk between these two structures. New evidence suggests that subchondral bone lesions like bone marrow edema and angiogenesis, can appear before cartilage degeneration. Therapeutics aimed at the subchondral bone have shown potential, meanwhile other treatments targeting cartilage have not had promising results. Abnormal subchondral bone remodeling, angiogenesis and sensory nerve innervation appear to contribute in different ways to cartilage destruction and pain [95]. Furthermore, damage of this interface has been proven to cause joint remodeling and angiogenesis, creating an opportunity for therapeutics targeting bone remodeling with agents such as bisphosphonates, calcitonin, TGF-β inhibitors, osteoprotegerin, VGEF antibody, Angiogenesis inhibitors, NGF antibody, mTOR inhibitors, CRISPR/Cas9 system for local ablation of NGF anti-cathepsin K, and bone-forming agents such as parathyroid hormone (PTH) [61,95].

## 4. Ongoing Investigations in the Role of Innate Immunity in OA Progression

Current studies from this group of investigators employing both in vitro culture techniques and in vivo models of joint disease support both an important role for innate immunity in joint disease progression in horses as well as the opportunity for potential targets for immune, and therefore disease, modulation through implementation of regenerative therapies. Ongoing investigations include assessment of the interaction of commonly injected orthobiological therapies on synovial macrophages and fibroblast-like synoviocytes for enhancement of chondrogenesis and immunomodulatory cytokine secretion, optimization of bone marrow aspirate concentrate fractions for treatment of osteoarthritis, and innate immune activation of mesenchymal stromal cells with Toll-like and Nod-like receptor agonists to reduce inflammation and improve control of multi-drug resistant synovial infections. To illustrate the potential practical applications of this review and bibliographical references cited herein demonstrating the importance of the innate immune response in joint disease, relevance of ongoing studies is briefly described below.

First, further research has focused on identifying the cell populations in regenerative therapies most active in reducing joint inflammation and cartilage degradation. The overall goal of this work is to optimize bone marrow aspirate concentrate (BMAC) aspirates by purifying for the most robust anti-inflammatory cell subpopulation that will improve efficacy and minimize deleterious side effects of cellular therapies. This work has initially focused on cell populations within BMAC, where modulation of paracrine signaling resulting in reduction of inflammation and chondral defect organization to be critical to the clinical benefit seen in BMAC treated cartilage defect repair [96]. The impact of purified BMAC cell groups on immunological properties including in vitro suppression of cytokine release from T-cells and macrophages and pro-chondrogenic activity, as well as in vivo reduction of joint inflammation and improvement in gait parameters in murine models of osteoarthritis will be evaluated. These studies are anticipated to improve our understanding of components of biological therapies that contribute most to clinical and histological improvement of osteoarthritis, with high relevance to OA in equine patients and significant and immediate translational application to human patients suffering from the same disease processes.

Secondly, the rapid development of antimicrobial resistance in veterinary and human medicine has prompted advancement of novel therapeutic strategies to improve infection control. Mesenchymal stromal cells (MSC) express immunomodulatory and antimicrobial properties through paracrine recruitment of immune effector cells and antimicrobial peptide secretion. Pre-activation of human, canine and equine MSC with Toll-like receptor (TLR) agonists such as polyinosinic-polycytidylic acid (polyI:C) has been shown to enhance bacterial killing and increase bacterial clearance in rodent *Staphylococcal* biofilm infection models [97,98,99]. This group of collaborators built further on that work to demonstrate that intra-articular administration of TLR3 polyI:C activated mesenchymal stromal cell therapy improved outcomes in treatment of multidrug resistant septic arthritis in an equine model, with reduced quantitative bacterial counts and pro-inflammatory biomarkers in synovial fluid, improved imaging (ultrasound and magnetic resonance imaging) scores and more rapid normalization of clinicopathologic parameters both systemically and in synovial fluid. These findings further support a future role for mesenchymal stromal cell therapy in immune modulation of inflammation associated with synovial infection towards more effective treatment of joint infections.

Finally, further research from this group seeks to use relevant in vitro bioassays to directly compare the anti-inflammatory and immunomodulatory disease-modifying activity of orthobiologic therapies commonly used in equine practice. Specifically, we seek to determine the macrophage and synoviocyte response to regenerative therapy exposure by measuring key cytokines in culture medium following exposure and use next-generation sequencing techniques to identify unique potentially disease-modifying pathways activated in synovial macrophages after treatment with biological therapies. These studies are intended to begin to fill a critical gap in our understanding of the relative immunomodulatory properties of regenerative therapies commonly used in equine practice to treat musculoskeletal disease.

## 5. Clinical Impact and Conclusions

New information continues to emerge from OA research suggesting a key role for the innate immune system in the pathogenesis of arthritis [100,101]. The data reported in the literature have shown that OA is a progressive disease which involves macrophages, leading to macrophage-related inflammation and degradation of local cartilage [25]. It is apparent that low-level, sustained innate immunity in joints contributes to the development of progressive OA. Improved understanding of the role of the innate immune system in the pathogenesis of OA, particularly in early stages of disease, will undoubtedly lead to new immune modulatory approaches to manage disease progression and reduce OA symptoms. Importantly, targeting the pro-inflammatory cascade in OA may lead to development of novel therapeutic strategies focused on re-establishing immune homeostasis in the joint. The key cells in all these processes, monocytes and macrophages, are the most important target for these new therapeutic approaches, which may be used to decrease macrophage activation and direct repolarization to an M2 phenotype. On the basis of our clinical experience, more pre-clinical animal models and clinical trials are necessary to evaluate the role of immune cells such as macrophages as selective targets in earlier stages of OA in the prevention and treatment of the disease. The development and testing of biological therapies such as MSCs in equine patients prior to use in humans holds tremendous potential for short- and long-term translational benefits in combating OA.

## Data Availability

Not applicable.
